# Conflict within species determines the value of a mutualism between species

**DOI:** 10.1002/evl3.109

**Published:** 2019-03-06

**Authors:** Syuan‐Jyun Sun, Nicholas P. C. Horrocks, Rebecca M. Kilner

**Affiliations:** ^1^ Department of Zoology University of Cambridge Cambridge CB2 3EJ United Kingdom

**Keywords:** Conflict, cooperation, fighting, social behavior, social evolution

## Abstract

Mutually beneficial interactions between species play a key role in maintaining biodiversity and ecosystem function. Nevertheless, such mutualisms can erode into antagonistic interactions. One explanation is that the fitness costs and benefits of interacting with a partner species vary among individuals. However, it is unclear why such variation exists. Here, we demonstrate that social behavior within species plays an important, though hitherto overlooked, role in determining the relative fitness to be gained from interacting with a second species. By combining laboratory experiments with field observations, we report that conflict within burying beetles *Nicrophorus vespilloides* influences the fitness that can be gained from interacting with the mite *Poecilochirus carabi*. Beetles transport these mites to carrion, upon which both species breed. We show that mites help beetles win intraspecific contests for this scarce resource: mites raise beetle body temperature, which enhances beetle competitive prowess. However, mites confer this benefit only upon smaller beetles, which are otherwise condemned by their size to lose contests for carrion. Larger beetles need no assistance to win a carcass and then lose reproductive success when breeding alongside mites. Thus, the extent of mutualism is dependent on an individual's inability to compete successfully and singlehandedly with conspecifics. Mutualisms degrade into antagonism when interactions with a partner species start to yield a net fitness loss, rather than a net fitness gain. This study suggests that interactions with conspecifics determine where this tipping point lies.

**Impact Summary**

Mutualisms are mutually beneficial interactions between species. They are widespread, are an important source of evolutionary innovation, and play a key role in maintaining biodiversity and ecosystem function. However, mutualisms reflect a delicate balance between the fitness benefits derived by associating with a partner species and the fitness costs associated with maintaining this partnership. They persist while the balance yields a net fitness gain but degrade when the balance tips to yield a net fitness loss, and one partner starts to exploit the other. A major outstanding challenge is to identify the factors that contribute to this tipping point to explain why interactions between two partner species are sometimes mutualistic, yet sometimes become more exploitative. In this article, we propose, and demonstrate experimentally, that social behavior *within* species can tip the balance from mutualism to antagonism.

Competition within species, for a limited resource or for a mate, means that some individuals are systematically placed at a disadvantage because they consistently lose to a rival. We hypothesize that it is these individuals that have the most to gain from entering into partnership with a second species, because the fitness benefits they stand to gain from a mutualistic partnership could potentially compensate any fitness lost through interactions with a conspecific rival.

We test this hypothesis by analyzing the relationship between burying beetles *Nicrophorus vespilloides* and the mite *Poecilochirus carabi*. These mites rely entirely on beetles for transport to small carrion, upon which they both breed. However, beetles face competition for carrion with rival conspecifics. Neither species can breed unless they secure this key resource.

We show that (1) mites help beetles win contests for carrion by raising beetle body temperature; (2) mites confer this benefit only on smaller beetles, which are otherwise doomed to lose fights against larger rivals. By contrast, larger beetles can secure a carcass without any help from mites; (3) mites help smaller beetles to produce more larvae because these beetles would not breed at all without the mites’ help. However, mites cause larger beetles to produce fewer larvae, because the mites compete for resources with beetle larvae on the carrion. Thus, mites are in a mutualism with smaller beetles but are parasites on larger beetles.

Mutualisms arise when two species cooperate to promote each other's fitness (Bronstein 2015). They are widespread, are an important source of evolutionary innovation, and play a key role in maintaining biodiversity and ecosystem function (Bronstein 2015). Mutualisms reflect a delicate balance between the fitness benefits derived by associating with a partner species and the fitness costs associated with maintaining this partnership. They persist while the balance yields a net fitness gain but are known to degrade when the balance tips to yield a net loss, and one partner starts to exploit the other (Palmer et al. 2008; Bronstein 2015; Hoeksema and Bruna 2015; Barker and Bronstein 2016). A major outstanding challenge is to identify the factors that contribute to this tipping point to explain why interactions between two partner species are sometimes mutualistic, yet sometimes become more exploitative.

Here, we propose and test the suggestion that social interactions *within* species can tip the balance from mutualism to antagonism. Social interactions within species are important in this regard because they establish variation in the fitness benefits that might be gained from interacting with a second species. Conflict with rivals (Emlen 2014), between the sexes (Kokko and Jennions 2014), or between parents and offspring (Hinde et al. 2010) means that some individuals consistently lose fitness after a social interaction. Even when individuals cooperate, the fitness benefits are seldom distributed equally between the social partners (Clutton‐Brock 2016). Social interactions with conspecifics thus place some individuals at a sustained fitness disadvantage, which potentially can be compensated through a mutualistic interaction with a second species. Mutualisms could arise if a second species directly induces a more favorable outcome from interactions with conspecifics (De Gasperin and Kilner 2015), or if it provides a service that compensates any fitness lost to conspecifics (Koide and Dickie 2002). Either way, the outcome of social interactions with conspecifics potentially explains variation in the extent of mutualism between species: those systematically disadvantaged by their conspecifics can potentially gain more from interacting with a second species.

We tested this idea by studying the burying beetle *Nicrophorus vespilloides* and its phoretic mite *Poecilochirus carabi*. Burying beetles (*Nicrophorus* spp.) use small vertebrate carrion as a resource for breeding, and there is competition for carcasses both within and among species. The ability to secure a carcass resource strongly determines a burying beetle's reproductive success (Scott 1998). This is particularly the case for females that, unlike males, require a carcass for reproduction, and cannot breed successfully without one. Competition among *Nicrophorus* spp. is sufficiently intense that it has likely caused character displacement, resulting in partitioning of the carrion niche among *Nicrophorus* spp. (Anderson 1982). Nevertheless, individuals still face density‐dependent competition for a carcass from rival conspecifics (Scott 1998). Beetles gain ownership of a carcass by wrestling, biting, and chasing competitors away (Supporting Information Video 1; see also Sun et al. 2014). Contests take place within each sex, and they are most likely to be won by larger beetles (Otronen 1988). Contests within *N. vespilloides* thus magnify individual size‐related variation in reproductive success (Steiger 2013; Pascoal et al. 2018), creating winners that are assured a high level of reproductive success after securing a carrion breeding resource, and losers that are much less likely to gain any reproductive success at all (Müller et al. 2007).

We determined whether this socially induced variation in prospective fitness influenced the extent of mutualism between *N. vespilloides* and the mite *P. carabi* (see Methods section). These mites feed and reproduce on carrion, just like burying beetles. However, whereas burying beetles can fly and search for small dead vertebrates, mites rely entirely on their beetle hosts to transport them to a carcass (Fig. S1). There they breed alongside the beetle using the same carrion resources for reproduction. Mites derive no nourishment from their hosts while they are passengers on the beetle, which is why they are described as phoretic, rather than parasitic. During reproduction on the carrion, however, beetle–mite interactions vary from mutualism to parasitism, depending on which family member's fitness is analyzed (De Gasperin and Kilner 2015) and on ecological factors such as the size of the carcass (De Gasperin and Kilner 2015) and the presence of rival blowflies (Bartlett 1988). Previous studies have focused entirely on interactions between mites and beetles after a carcass has been secured. We investigated whether mites could assist burying beetles in obtaining a carcass for both species to breed upon by enhancing the beetle's competitive prowess.

## Methods

### STUDY SPECIES

Burying beetles *N. vespilloides* (hereafter simply “beetles”) use small carrion to breed upon, such as a dead mouse or songbird. They prepare the dead body for their own reproduction by removing the fur or feathers and rolling the carcass into a ball (De Gasperin et al. 2016), which they bury below ground. The larvae hatch from eggs laid in the soil nearby and crawl to the carcass. There they take up residence on the edible carrion nest, where they feed themselves and are fed by their parents. Parents leave the breeding attempt between 1 and 5 days after hatching, flying off to seek new reproductive opportunities (De Gasperin et al. 2015). Roughly 8 days after the parents first located the carcass, larvae start to disperse away into the surrounding soil to pupate. Two to three weeks later, the larvae emerge as adults and after another two weeks they are sexually mature.

Beetles transport mites to breed on the carrion. There are about 14 species of mites associated with burying beetle species, belonging to four different families associated with *Nicrophorus* beetles (Wilson and Knollenberg 1987). *Poecilochirus carabi* (*Arachnida: Acari*) is the most common of these mite species (around 95% of the mites found on beetles in nature are *P. carabi* (Schwarz et al. 1998)). It exists as a species complex, consisting of races that are each specialized on a different *Nicrophorus* species (Nehring et al. 2017). Multiple races can be found on one beetle (Nehring et al. 2017) but they cannot be distinguished morphologically. Our unpublished behavioral data suggest that the mites used in these experiments (hereafter simply “mites”) comprised a mixture of the *N. humator* and *N. vespilloides* races.

*P. carabi* mites travel as deutonymphs on the burying beetles (Fig. S1). Once carrion has been located, they disembark from the beetle, molt into adults in the soil next to the carcass, and start breeding by using resources on the carrion. The presence of a carcass is essential for mites to both molt and breed successfully. The next generation of deutonymphs mostly disperses with the adult beetles as they fly off after reproduction (Schwarz and Koulianos 1999).

All beetles and mites used in the experiments were descendants from a field‐caught population collected in Madingley Woods, near Cambridge, UK (latitude: 52.22658°; longitude: 0.04303°) between August and October 2016. They were brought into the laboratory, in the Department of Zoology at the University of Cambridge, UK, and maintained on a 16:8 h light:dark cycle at 20°C.

### BURYING BEETLE HUSBANDRY

To breed burying beetles, pairs of unrelated males and females were placed in plastic breeding boxes two to three weeks after eclosion (17 × 12 × 6 cm filled with 2 cm depth of moist soil) and provided with a mouse carcass (bought commercially from Livefood UK Ltd., Axbridge, UK). The box was placed in a cupboard to simulate underground conditions. Eight days after pairing, as larvae were starting to disperse away from the carcass, we collected the larvae and transferred them to eclosion boxes (10 × 10 × 2 cm, 25 compartments) filled with damp soil, with one larva occupying each cell. Approximately three weeks later, after eclosion, each beetle was placed by itself in a plastic container with soil (12 × 8 × 2 cm) and fed with beef mince twice a week until it was used in the experiments.

### MITE HUSBANDRY

To provide a continual source of mites for our experiments, we started by providing pairs of burying beetles with a mouse carcass in a breeding box, as described above. Fifteen mite deutonymphs, randomly drawn from different individual beetles collected in different traps, were introduced to breed alongside the beetles (*n* = 10 breeding pairs). At the end of each breeding event, the next generation of mite deutonymphs was collected as it dispersed on beetle adults, using CO_2_ anaesthetization. Once separated, mites were kept alongside an adult beetle in a breeding box, fed with beef mince twice a week. We replenished the mite population each month by again placing laboratory‐bred mites with breeding pairs of beetles (*n* = 10 breeding pairs) and allowing mites to breed.

### NATURAL POPULATIONS

To determine the distribution of *N. vespilloides* body size in nature, and the number of mites typically carried by each individual, we trapped beetles between June and October in 2017, which covers the entire breeding season at our study site. Traps, baited with either mice or chick carcasses, were set up constantly during this time in Gamlingay (latitude: 52.15555°; longitude: −0.19286°), Waresley (latitude: 52.17487°; longitude: −0.17354°), and Madingley (latitude: 52.22658°; longitude: 0.04303°) Woods in Cambridgeshire, UK. They were all checked every two weeks. Beetles were collected and brought back to the laboratory in the Department of Zoology. There they were anaesthetized with carbon dioxide prior to the collection of body measurements and removal of mites. The body size of every beetle was recorded by measuring pronotum width to the nearest 0.01 mm (this is a standard way to measure adult beetle size, see also Jarrett et al. 2017) and the total number of mites on each beetle was also recorded.

In total, 1369 live beetles were trapped. The size distribution of these beetles is shown in Figure S2. This was used to define beetles as “small,” “medium,” and “large” for use in our experiments. Note that we have previously shown that the heritability of burying beetle body size does not differ significantly from zero (Jarrett et al. 2017). Instead, variation in body size depends on the extent to which larvae are nourished during their development (Lock et al. 2004). Hence body size is effectively reset environmentally at each generation, and selection against smaller individuals is not sufficient to cause the evolution of increased body size within a population. From Figure S2, we have *n* = 340 for small beetles (pronotum width <4.43 mm) and *n* = 360 for large beetles (pronotum width >5.08 mm).

Wild‐caught beetles differed in the number of mites they carried, but only 124 beetles (9%), carried more than 30 mites (dashed line; Fig. S3a; see below for why this number is relevant). Large beetles generally carried more mites than small beetles (generalized linear mixed model [GLMM], *χ*² = 29.06, df = 1, *P <* 0.001; Fig. S3b). The mean numbers of mites carried by small and large beetles, respectively, were 8.09 ± 0.56 and 14.34 ± 1.32. Of these beetles, 19 (5.59%) smaller beetles carried 30 mites or more, whereas 47 larger beetles (13.06%) carried 30 mites or more. Thus, larger beetles were more likely than smaller beetles to bear loads of at least 30 mites (GLMM, *χ*² = 10.18, df = 1, *P =* 0.001). Beetles do not appear to be able to control the number of mites that they carry (S.‐J. Sun, unpubl. data), and nor do mites seem to preferentially associate with other mites, but there does appear to be a preference among mites to associate with large beetles over small beetles (S.‐J. Sun and N.P.C. Horrocks, unpubl. data). This may explain the skew in mite distribution that we observe with beetle size.

## Experiments

### EFFECT OF MITES AND BEETLE BODY TEMPERATURE ON CONTESTS BETWEEN RIVAL FEMALES

#### Treatments

We staged contests between beetles in three different ways: (1) a beetle with mites versus a beetle without mites (*n* = 23 contests); (2) a beetle without mites versus a beetle without mites warmed up (*n* = 20 contests) to simulate beetle body temperature if mites were present (“warmed” beetle), and (3) a beetle with mites versus a beetle with mites cooled down (*n* = 20 contests) to simulate body temperature if mites were not present (“cooled” beetle). In all contests, the contestants were two females, two to three weeks posteclosion, and matched in body size using measurements of their pronotum width to 0.01 mm (mean ± SE = 0.0095 ± 0.0010 mm). This minimized any effects of body size on contest outcome so that we could investigate effects that were due to mites and body temperature. Before the experiment females were virgins, but just prior to each contest they were mated with unrelated males, because females are typically already mated when they locate a carcass in nature. Contestants were each marked with Testors enamel paint (Butler et al. 2012) on the elytra before the fight, for easy identification. Each beetle was only used once in a single contest. For beetles treated with mites, we introduced 30 mites to beetles 30 min before contests began (see below for explanation of why we chose this mite density). Beetles that were experimentally warmed or cooled were in incubators set to 21.5°C (warmed beetles) and 18.5°C (cooled beetles), for 30 min prior to each trial. All contests took place at an ambient temperature of 20°C. Each contest was staged in a plastic container (28.5 × 13.5 × 12 cm) containing 2 cm depth of soil and a dead mouse (8–13 g). Previous studies have shown that *N. vespilloides* arriving earlier at carcasses are more likely to win any ensuing contest (Otronen 1988). Therefore, to prevent any possible confounding effects of prior arrival, the contestants were placed simultaneously in the contest arena. During each contest, individuals were able to leave the arena via a one‐way valve (see De Gasperin et al. 2015 for further details).

#### Behavioral observations

A USB camera powered by a PC, with a resolution of 1920 × 1080 pixels, was used to record any aggression that occurred in the first 30 min of each trial. We classified aggressive acts as wrestling, biting, or chasing of one individual by the other (Sun et al. 2014). At the end of filming, we continued to observe the contest for the next 3 h, scanning the arena every 30 min to determine the outcome. When only one beetle remained on the carcass for two consecutive observations, she was deemed to be the winner. Unfortunately, we did not record the number of aggressive acts in beetles that were heated or cooled, and so further studies are required to determine the relative influences of aggression and body temperature on the likelihood of victory.

#### Infrared thermography

Contests were also filmed with a FLIR T460 infrared camera (thermal sensitivity: <0.03°C at 30°C, 2% accuracy at 25°C) at a resolution of 320 × 240 pixels with frame rate at 30 fps. Using the software FLIR Tools+ 6.4 (Copyright 2018 FLIR Systems, Inc; http://www.flir.com), the body temperature of beetles was measured at the center of the thorax, and the temperature of soil was determined as the average temperature measured inside a 2 cm diameter circle randomly oriented adjacent to where a beetle was sitting on the soil. Throughout the study (but see treadmill experiments below for exception), all beetle body temperatures are therefore presented as the difference between these two measurements. We determined the emissivity of beetle cuticle using methods described in the Supporting Information of Smolka et al. 2012. To ensure accurate measurement of temperature, all measurements were taken at a constant distance of 25 cm from the surface being measured. The calibrated infrared emissivity of beetle and soil was set to 0.95 (0.947 ± 0.02, *n* = 23); in this way, all measures were scaled in relation to the thermal radiation emitted by a black body. We synchronized footage from the infrared camera with our standard film of the contest to determine beetle body temperature and soil temperature 2 sec before a fight started and 2 sec after a fight ended.

### EFFECT OF MITE DENSITY ON BEETLE BODY TEMPERATURE

We measured female body size by measuring the width of her pronotum. We then added groups of 10 mite deutonymphs sequentially to the same individual female. Thus, each female started with zero mites, then had 10, then 20, and finally 30 mites added (*n* = 45 beetles). To control for the potential order effects of mite association, we also manipulated mite number in reverse order by initially adding 30 mites and then removing 10 mites at a time (*n* = 45 beetles). We measured beetle body temperature 30 min after the addition, or removal, of 10 mites, using the infrared camera described above (Fig. S4). The effect of mite density on beetle body temperature, in relation to beetle body size, is shown in Figure S5.

### ARE MITES A SOURCE OF HEAT?

To determine whether changes in beetle body temperature were due to mites or beetles, we added or removed mites in batches of 10 in exactly the same way as described above (adding mites: *n* = 10 beetles; removing mites: *n* = 10 beetles), but this time using dead female beetles. The dead beetles were killed just before the experiment by exposing them to –20°C for 1 h. They were then put in an incubator at 20°C until they acclimated to environmental temperature in the laboratory. At this point, they were used in the experiment.

### TREADMILL EXPERIMENTS

To determine whether the beetle's elevated body temperature was due to carrying the weight of mites and/or whether mites serve as an insulating blanket, we continuously measured changes in beetle body temperature using infrared thermography across time for small (4.05 ± 0.044 mm, *n* = 17) and large (5.21 ± 0.030 mm, *n* = 17) beetles, as they walked on a motorized treadmill modified from a laboratory tube rotator (Supporting Information Video 2). Beetles walked on the continuously moving track for 1 min. Preliminary tests showed that walking for this amount of time produced an increase in beetle body temperature of approximately 1°C, which we interpreted as evidence that the beetles were doing considerable work (for comparison, beetle body temperature increased by only about 0.2–0.3°C in beetles that had engaged in a contest over a carcass; Fig. 2). The track was set to move at a constant speed of 4.7 cm sec^−1^ (see Supporting Information Video 2), which is the slowest walking speed of a beetle carrying no mites (mean speed, 6.74 ± 0.476 cm sec^−1^; *n* = 13 beetles). This period of exercise was followed by a 3‐min rest phase, the duration of which was chosen because previous work has shown that beetle body temperature can decline dramatically postexercise in this timeframe (Merrick and Smith, 2004). Beetle body temperature was measured at the center of the thorax every 10 sec and 20 sec during walking and resting phases, respectively. We tested each beetle three times, randomly mixing the sequence in which they carried the following loads: (1) control—no load, (2) 30 mites (7.78 ± 0.307 mg), and (3) an experimental weight, equivalent to 30 mites (8.04 ± 0.101 mg). The experimental weight consisted of a blob of Blu‐Tack® that was gently molded and attached to the front portion of the elytra, which is where *P. carabi* mites are typically located (Fig. S1). This allowed us to temporarily manipulate the body mass of beetles without causing trauma. Mean body masses for small and large beetles, respectively, were 120.99 ± 3.92 mg and 222.44 ± 3.87 mg. Thus, carrying mites increased the body mass of small and large beetles by 6.5% and 3.6%, respectively (*t* test, *t* = 5.33, *P* < 0.001), whereas carrying experimental weights increased the body mass of small and large beetles by 6.7% and 3.7%, respectively (*t* test, *t* = 8.11, *P* < 0.001).

### MEASUREMENT OF BEETLE SURFACE AREA, SURFACE AREA COVERED BY MITES, AND SURFACE AREA:VOLUME RATIO

To further understand how mites were able to insulate beetles, we estimated a beetle's surface area, the area covered by different numbers of mites, and the surface area:volume ratio of small (4.08 ± 0.101 mm, *n* = 11) and large (5.22 ± 0.043 mm, *n* = 10) beetles. We added groups of 10 mites, up to a total of 30 mites, to the same individual female, or else added 30 mites, and then removed 10 mites at a time, to control for the order effects of manipulation. After addition or removal of mites, we allowed 10 sec for the mites to freely distribute themselves over the surface of the beetle. The ventral and dorsal surface of every beetle was then photographed next to a scale bar at a constant distance and under the same lighting conditions. We similarly took photos of beetles carrying an experimental weight equivalent to 30 mites (see details in Treadmill experiments section). All digital images were analyzed using image analysis software (ImageJ, https://imagej.nih.gov/ij/). We extracted data from calibrated images by calculating the area of the dorsal and ventral surfaces, the area covered by 0, 10, 20, or 30 mites, and the area covered by the experimental weight. For simplicity, we assumed that beetles are two‐dimensional, meaning we could estimate total body surface area as the sum of the area of the dorsal and ventral views. After photography, beetles were euthanized by exposing them to –20°C for 1 h, all mites were removed, and the volume of each beetle was determined by the water displacement method. Because we were interested in understanding how the presence of mites influenced the potential for heat loss, we calculated surface area:volume ratios as the ratio of uninsulated beetle surface area (sum of the beetle surface area – sum of the surface area of *n* mites) divided by beetle volume.

We began by checking that our methods were not confounded by only taking measurements from the dorsal and ventral surfaces of beetles. This is because some mites were present on the lateral surfaces of beetles, but they were not included in our estimation of the beetle surface area covered by mites. To determine whether or not this was a potential confounding effect, we counted the number of mites that attached to the sides of beetles and tested whether the number differed with the mite density on the beetle (10, 30, 30 mites), the beetle's size (small/large), and the interaction between mite treatment and beetle size. Neither the interaction term (*χ*² = 2.00, df = 2, *P* = 0.368) nor beetle size (*χ*² = 1.54, df = 1, *P* = 0.214) significantly influenced the number of mites that were found on the lateral surfaces of beetles, although we did find more beetles on lateral surfaces at greater mite densities (*χ*² = 15.74, df = 2, *P* < 0.001). This means we have underestimated the surface area covered by mites, particularly at high mite densities. Therefore, the results of the analyses that follow are biased to be conservative.

We found that 30 mites covered a greater proportion of the surface area of a small beetle than they did on a large beetle (22.3% and 8.8%, respectively; GLMM, beetle size × mite number interaction: *χ*² = 75.59, df = 3, *P <* 0.001; Fig. S6a). Furthermore, relative to the beetle's volume, 30 mites covered a greater surface area on small beetles than did any other mite load, leaving a much smaller area exposed and therefore uninsulated (GLMM, beetle size × mite number interaction: *χ*² = 35.93, df = 4, *P <* 0.001; Fig. S6b). These results explain why we found a nonlinear relationship between mite number and beetle body temperature (Fig. S5).

By contrast, the experimental weight had no insulative properties (Fig. 3C), because it covered a much smaller surface area. Nevertheless, it occupied proportionately more surface area on a small beetle than on a large beetle (small beetles: *t*‐ratio = 14.25, *P* < 0.001; large beetles: *t*‐ratio = 3.34, *P* = 0.030; Fig. S6a). For large beetles, whether beetles carried 30 mites, or the experimental weight, or no mites at all, there was no significant change in the surface area that was exposed, relative to the beetle's volume (30 mites: *t*‐ratio = 2.54, *P* = 0.266; experimental weight: *t*‐ratio = 1.71, *P* = 0.787; Fig. S6b). This may explain why rate of heat loss in all three treatment groups for large beetles was essentially the same (Fig. 3C).

### EFFECT OF MITES ON CONTESTS WHEN BEETLES DIFFER IN SIZE

We again staged contests between two females over a dead mouse, but this time we ensured that the contestants differed in size. In one treatment, focal beetles were small (4.29 ± 0.019 mm, *n* = 40), whereas in a second treatment focal beetles were large (5.14 ± 0.026 mm, *n* = 37). In each contest, focal beetles were pitted against a different medium‐sized beetle (4.67 ± 0.011 mm; Fig. S2). In roughly half the contests, the focal beetle bore 30 mites, which were added beforehand as described above (*n* = 20 small beetles, *n* = 19 large beetles). In all other details, the procedure for staging the contest was exactly as described above, except that we did not film these contests.

### EFFECT OF MITES ON BURYING BEETLE REPRODUCTIVE SUCCESS, WITH RESPECT TO BEETLE BODY SIZE

We determined the effects of mites on reproductive success by breeding small (4.20 ± 0.024 mm, *n* = 67) and large (5.17 ± 0.021 mm, *n* = 61) females with either 0 or 30 mites on 8–13 g mouse carcasses (9.51 ± 0.093 g, *n* = 128). For the treatment with mites, 30 deutonymphs were added as we introduced females to breeding carcasses. These beetles did not experience a contest prior to breeding. At dispersal, we counted and weighed all dispersing third‐instar larvae as a proxy of breeding success.

### EFFECT OF MITE DENSITY ON MITE REPRODUCTIVE SUCCESS

To investigate how mite density influences mite deutonymphs’ molting rate, we repeated the experiments described in Nehring and Müller (2009). Mites were kept as groups of 1 (*n* = 19), 2 (*n* = 22), 4 (*n* = 22), 8 (*n* = 18), 10 (*n* = 20), 12 (*n* = 20), 16 (*n* = 19), 20 (*n* = 19), or 30 (*n* = 20), on pieces of moist filter paper within Petri dishes (diameter 60 mm, height 15 mm). Each group was provided with a piece of lamb liver (0.6–0.8 g) to trigger molting. After two days, we checked the number of deutonymphs that molted into males or females or remained unmolted.

## Statistical analyses

All analyses were conducted in R version 3.4.3 (R Development Core Team 2014). GLMMs were used in the package *lme4* (Bates et al. 2015) with fixed and random factors to analyze the effects of mite number on body temperature and the contest outcomes.

### EFFECT OF MITES AND BEETLE BODY TEMPERATURE ON CONTESTS BETWEEN RIVAL FEMALES

To examine the contest outcomes of each of the three experimental treatments (i.e., with mites vs. without mites, without mites/warmed vs. without mites, and with mites/cooled vs. with mites), we performed GLMMs with a binomial distribution by including mite treatment, carcass mass, and relative difference in body size, that is, [(focal female pronotum width – non‐focal female pronotum width)/focal female pronotum width] as fixed factors. To investigate mite effects on body temperature 2 sec before each aggression, we included mite treatment, carcass mass, and relative difference in body size as fixed factors. Each contest consisted of a single outcome (win or lose), but within each contest multiple aggressive acts could be recorded for each beetle. Therefore, we included order of the aggressive act (i.e., first aggressive act, second aggressive act, and so on) nested within ID of each trial as a random factor. Because we had no a priori expectation as to how mites or temperature should affect beetle aggressive behavior, we grouped all behavior types (wrestling, biting, or chasing) together in our analyses, while still recording the total number of aggressive acts occurring within a contest. For each act of aggression that occurred between beetles within a contest, we also analyzed temperature differences 2 sec after the act of aggression for beetles with and without mites. For this analysis, we included the interaction between temperature differences before the fight and mite treatment, carcass mass, and relative body size difference as fixed factors, and order of the aggressive act nested within trial ID as a random factor. We included “block” as a fixed factor, because the experiment was carried out with beetles from two consecutive generations.

### EFFECT OF MITE DENSITY ON BEETLE BODY TEMPERATURE

To examine how mite number affected body temperature, we included the difference between body temperature and soil temperature as a dependent variable, and mite number treatment (0, 10, 20, and 30 mites as a categorical variable), sequence of mite association (increase or decrease), and body size of each individual as fixed factors, and individual ID as a random factor.

To further understand the effects of mites on the proportional increase in temperature across body size, temperature difference and the ratio of temperature difference (dividing temperature difference with mites by temperature difference without mites) were included as dependent variables. Mite number, body size, and their interaction, and order of mite association were included as fixed factors, and individual ID as a random factor.

### ARE MITES A SOURCE OF HEAT?

To examine whether mites themselves generate heat, we analyzed the difference between body temperature and soil temperature for 10 freshly killed beetles in the same way as described above.

### TREADMILL EXPERIMENTS

To assess whether the equivalent weight of mites differentially affects the body temperature for small and large beetles as they walk on a treadmill, we analyzed the interacting effects between treatments (control, mite, and weight) and body size (small or large) across the walking period (0–60 sec) on the temperature difference (focal body temperature – body temperature at time 0 sec; note that this way of measuring body temperature is slightly different to the method used in all other experiments). Focal body temperature was sampled every 10 sec. To assess whether mites provide an insulative “blanket effect” to reduce beetles’ heat loss while resting after walking, we analyzed the interacting effects between treatments (control, mite, and weight), body size (small or large), and time (60–240 sec) on the temperature difference (focal body temperature – body temperature at time 60 sec; again, for logistical reasons, body temperature is measured slightly differently to the method used in all other experiments). Focal body temperature was sampled every 20 sec. For both analyses, individual ID was included as a random factor as individual beetles were repeatedly sampled.

### MEASUREMENT OF BEETLE SURFACE AREA, SURFACE AREA COVERED BY MITES, AND SURFACE AREA:VOLUME RATIO

We analyzed whether carrying mites or the experimental weight changed the proportion of surface area exposed, and whether this differed between small beetles and large beetles. In this analysis, the dependent variable was the proportion of beetle surface area covered by mites (or the experimental weight), calculated as the sum of the surface area covered/sum of the dorsal and ventral surface areas. This measure was log‐transformed prior to analysis to meet assumptions of data normality. We included the load borne by the beetle (i.e., 10, 20 or 30 mites, or the experimental weight) and body size (small or large) as fixed factors, and also the interaction between them. Individual ID was included as a random factor as individuals were repeatedly sampled. We used a similar model to determine how these variables influenced a beetle's surface area, relative to its volume. The only difference was that in this model the dependent variable was calculated as the (sum of the beetle surface area – sum the surface area covered by mites)/beetle volume.

### EFFECT OF MITES ON CONTESTS WHEN BEETLES DIFFER IN SIZE

We analyzed the effects of mites on the outcome of a contest when beetles differed in body size using a binomial distribution. The outcome (winner or loser) was included as a dependent variable, while mite treatment, body size (small or large), carcass mass, and relative difference in body size were included as fixed factors.

### EFFECT OF MITES ON BURYING BEETLE REPRODUCTIVE SUCCESS, WITH RESPECT TO BEETLE BODY SIZE

We analyzed the effects of mites on brood size at dispersal, for small and large beetles, by using a Poisson distribution and including mite treatment and carcass mass as independent variables.

### EFFECT OF MITE DENSITY ON MITE REPRODUCTIVE SUCCESS

To test for an influence of mite density on the molting rate of mites, we analyzed the effect of number of mites (as a continuous predictor) on the proportion of molted mites using a GLM (generalized linear model) with a binomial distribution and a logit link function. The model that fitted best was a second‐order polynomial regression (*χ*² = 101.59, df = 1, *P <* 0.001; Fig. S7), replicating the findings of a previous study (Nehring and Müller 2009). We also checked whether there was an effect of the amount of liver that was provided to the mites on the likelihood of molting. Liver mass had no effect on proportion of mites molting (*χ*² = 0.93, df = 1, *P =* 0.335).

## Results and Discussion

### DO MITES AFFECT THE OUTCOME OF CONTESTS BETWEEN BEETLES OVER CARRION?

**Figure 1:**
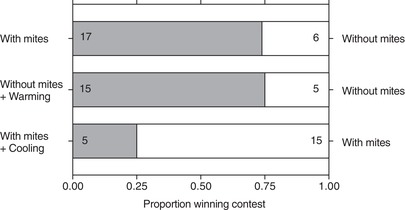
**The independent effects of mites and temperature on contest outcome**. Beetles were evenly matched for body size in all contests. Numbers indicate trials won by beetles.

We began by staging contests between rival female burying beetles for a carcass, loading one female with 30 mites while leaving her rival mite free (see Methods section). Female beetles were closely matched in body size so that we could attribute any variation in the outcome of a contest to the mites alone. At the start of each trial, females were placed simultaneously on a small mouse carcass and their interactions were filmed (Supporting Information Video 1). We found that females bearing mites were three times more likely to exhibit acts of aggression than beetles without mites (62 out of 80 aggressive behaviors recorded across all contests were initiated by beetles with mites; GLMM, *χ*² = 21.10, df = 1, *P* < 0.001). Furthermore, beetles with mites were also more likely to win contests over breeding carcasses (GLMM, *χ*² = 9.82, df = 1, *P* = 0.002; Fig. 1).

**Figure 2:**
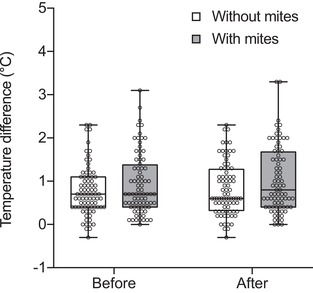
**The effect of mite association on body temperature 2 sec before and 2 sec after each contest**. The median, inter‐quartile range, and range of data are shown in the boxplots. Each boxplot shows data from 80 acts of aggression. “Temperature difference” refers to the difference between temperature of the beetle and the temperature of the soil it is on.

We then investigated whether the competitive superiority conferred by mites was associated with an elevated body temperature in the beetles, since this has been shown to influence competitive ability in other insects (Stutt and Willmer 1998). Infrared thermography revealed that beetles bearing mites had a higher body temperature before acts of aggression than mite‐free beetles (beetle body temperature with mites = 0.941 ± 0.078°C; without mites = 0.766 ± 0.063°C: GLMM, *χ*² = 9.72, df = 1, *P =* 0.002;. Fig. 2 and Supporting Information Video 1). Fighting caused all beetles to raise their body temperature, but this increase was much greater for beetles with mites compared to beetles without mites (beetle body temperature with mites = 1.038 ± 0.086°C; without mites = 0.823 ± 0.068°C: GLMM, temperature difference before fighting × mite treatment: *χ*² = 7.61, df = 1, *P =* 0.006; Fig. 2).

### ARE HOTTER BEETLES MORE LIKELY TO WIN CONTESTS, INDEPENDENT OF MITES?

We next determined whether a raised body temperature was sufficient to improve success at winning contests, independent of an association with mites. We followed the same protocol as before, staging contests over a dead mouse between rival females that were matched in size (see Methods section). This time, neither female carried mites. Instead, prior to a contest, one of the females was placed in an incubator at 21.5°C for 30 min. This increased body temperature by 1.15 ± 0.14°C compared to the rival female that was not heated, generating a similar temperature difference (1.04 ± 0.17°C) between beetles with and without mites to that seen in the first experiment (GLM, *χ*² = 0.02, df = 1, *P =* 0.892). The elevated body temperature increased the likelihood of this female winning the contest (*n* = 20 contests; GLMM, *χ*² = 9.30, df = 1, *P* = 0.002), with a success rate that was very similar to that induced by mites (Fig. 1). In 20 further contests, rival females each bore mites but one was cooled beforehand by placing her in an incubator at 18.5°C for 30 min. This reduced body temperature by 1.07 ± 0.17°C compared to the rival uncooled beetle. Experimental cooling also reduced the probability of winning a contest (GLMM, *χ*² = 8.98, df = 1, *P* = 0.003; Fig. 1), even though the losing female bore mites. We conclude from these experiments that mites enhance burying beetle competitive prowess by raising the beetle's body temperature; the presence of mites alone is not sufficient to guarantee victory in a fight.

### HOW MANY MITES ARE REQUIRED TO RAISE BEETLE TEMPERATURE SUFFICIENTLY TO WIN A CONTEST?

In natural populations, there is considerable variation in the number of mites carried by individual beetles, ranging from 0 to 285 per beetle (see Methods section; Fig. S3a). We analyzed how the mite density on a burying beetle influenced its body temperature by adding different numbers of mites: 0, 10, 20, or 30 mites (91% beetles carry 0–30 mites in natural populations; Fig. S3a). We found a nonlinear relationship between mite density and beetle body temperature, with a beetle's temperature rising sharply when it carried more than 20 mites (GLMM, *χ*² = 112.04, df = 3, *P* < 0.001; Fig. S4). Adding 30 mites caused a rise in temperature (1.598 ± 0.090°C) that was similar to that induced by the incubator in the previous experiment (GLM, *χ*² = 0.67, df = 1, *P* = 0.414).

### DO MITES WARM SMALLER AND LARGER BEETLES TO THE SAME EXTENT?

We found that larger beetles were warmer than smaller beetles, even without mites (GLMM, body size effect: *χ*² = 20.18, df = 1, *P* < 0.001), which might be due to their relatively smaller surface area‐volume ratio (SA/V), and consequently lower expected rates of heat loss than smaller beetles (Stutt and Willmer 1998). Their consistently greater body temperature could explain, in part, why larger beetles so frequently win fights with conspecifics. The effect of the mites on beetle body temperature also varied with beetle size (GLMM, body size × mite number interaction: *χ*² = 20.15, df = 4, *P* < 0.001). Mites caused a proportionally greater increase in body temperature in smaller beetles than in larger beetles (Fig. S5), especially when 30 mites were added to the beetle (GLM, mite number effect: *χ*² = 29.40, df = 2, *P* < 0.001).

### HOW DO MITES WARM SMALLER BEETLES TO A GREATER EXTENT THAN LARGER BEETLES?

To determine whether mites themselves were generating heat, we compared the body temperature of freshly killed beetles with and without mites (see Methods section). We could not detect a difference in temperature between the two treatments (GLMM, presence vs. absence of mites: *χ*² = 1.73, df = 1, *P =* 0.188), suggesting that mites were not a source of heat themselves. Next, we tested whether mites cause beetles to generate heat, because they add to the mass borne by a beetle and increase the work involved in beetle locomotion. We also analyzed whether mites could act as an insulator and slow down the rate at which heat generated by beetles is lost. To test these ideas, we induced small and large female beetles to walk on a motorized treadmill (see Methods section; Supporting Information Video 2), while loaded with either 30 mites, or a weight equivalent to the mass of 30 mites, or bearing no load at all. Each beetle was tested with all three treatments, applied in random order across beetles. After 1 min of walking on the treadmill, beetles were allowed to rest for 3 min. We measured body temperature every 10 sec and 20 sec during the walking and resting phases, respectively (see Methods section).

**Figure 3:**
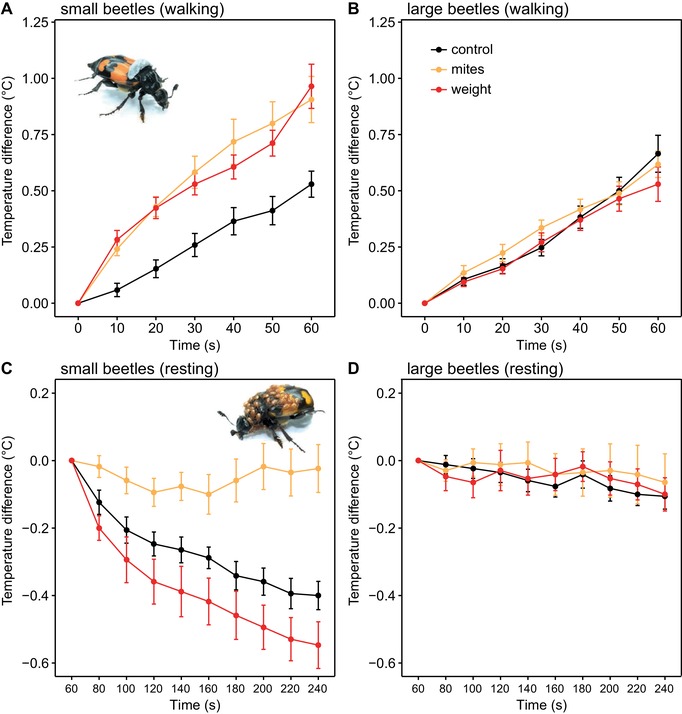
**The effect of mites on burying beetle body temperature during exercise and subsequent rest. “**Temperature difference” refers to the difference between the temperature of the beetle during the experiment and at the start of the respective experiment (see Methods section for more details). This is shown for the different loading treatments across time, during walking by (A) small beetles and (B) large beetles, and during the subsequent resting period for (C) small beetles and (D) large beetles. Inset images show the different loads borne by beetles in the different experimental treatments: (A) weight (C) and 30 mites*. n* = 17 for both small and large beetles.

The treadmill experiments revealed that there were interactions between the beetle size and loading treatments across the timing for both walking (walking: GLMM, beetle size × loading treatment × time interaction: *χ*² = 21.36, df = 12, *P =* 0.045) and resting (resting: GLMM, beetle size × loading treatment × time interaction: *χ*² = 32.98, df = 18, *P =* 0.017). Specifically, small beetles carrying mites, or weights of equivalent mass, attained a higher body temperature during locomotion than control beetles (Fig. 3a and Table S1), but there was no equivalent effect on the body temperature of large beetles (GLMM, loading treatment × time interaction: *χ*² = 6.17, df = 12, *P =* 0.907; Table S1). This is because the body temperature of larger beetles rose to a similar extent during walking on the treadmill, whether or not they were carrying anything (Fig. 3B). During the resting period, small beetles maintained a stable elevated temperature for longer when they carried mites than when they either bore a weight or were unencumbered (Fig. 3C and Table S1). By contrast, large beetles were able to maintain an elevated body temperature after locomotion without the addition of mites or weights (Fig. 3D and Table S1). These size‐related effects arise probably because 30 mites add proportionally greater mass to a small beetle than to a large beetle (see Methods section). Locomotion by smaller beetles correspondingly requires more power and elevates body temperature to a greater extent (Schmidt‐Nielsen 1997). Similarly, 30 mites cover a greater proportion of a small beetle's surface area (see Methods section; Fig. S6a), and also decrease to a greater extent the opportunity for heat loss through exposed body surface area (see Methods section; Fig. S6b). Thus, mites are more effective at reducing the rate of temperature loss on smaller individuals, but the thermal effects of mites on smaller beetles arise as a by‐product of riding as passengers on the beetle, and probably did not evolve specifically to assist burying beetles.

### DO MITES HELP SMALLER BEETLES WIN CONTESTS OVER CARRION: FITNESS BENEFITS OF THE PARTNERSHIP

**Figure 4:**
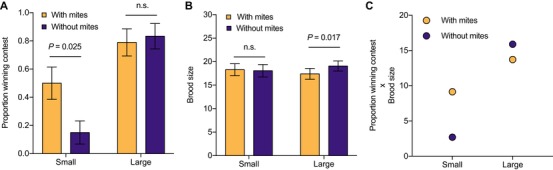
**The effects of mites on burying beetle fitness, in relation to the beetle's size**. (A) Proportion winning a contest against a medium‐sized female, in the presence or absence of mites (*n* = 40 small females and *n* = 37 large females). (B) Brood size at larval dispersal for small females and large females breeding in the presence or absence of mites (small females: *n* = 34 with mites and 33 without mites; large females: *n* = 33 with mites and 28 without mites). Means ± SE are shown. (C) Mean burying beetle fitness, calculated as the product of the mean probability of winning a contest (from A) and the mean number of larvae produced (from B), in relation to beetle size and the presence or absence of 30 mites.

We next investigated whether the mite‐induced thermal effects on smaller beetles are sufficient to compensate for the size‐related disadvantage they face during contests for a carcass. We pitted focal beetles against rival medium‐sized beetles (4.67 ± 0.011 mm) in contests over a dead mouse (see Methods section). Focal beetles were either small (4.29 ± 0.019 mm) or large (5.14 ± 0.026 mm) and were either loaded with 30 mites or bore no mites at all. Overall, we found that mites increased the likelihood that a smaller beetle would win the contest, but mites had no equivalent effect on larger beetles (GLM, mite × beetle size interaction *χ*² = 4.03, df = 1, *P =* 0.045; Fig. 4A). Small beetles with mites were almost three times more likely to win a contest over a carcass than were small beetles without mites (GLM, presence vs. absence of mites: χ² = 5.01, df = 1, *P =* 0.025; Fig. 4A). Large beetles were highly successful at winning contests even without mites: bearing mites did not change their chance of victory (GLM, presence vs. absence of mites: *χ*² = 0.32, df = 1, *P =* 0.574; Fig. 4A). Smaller loser beetles thus gain more from interacting with mites than larger winner beetles. Thus, social interactions within species determine the magnitude of the fitness benefit conferred by a second species.

### DO MITES REDUCE BURYING BEETLE BROOD SIZE: FITNESS COSTS OF THE PARTNERSHIP?

Having secured a carcass, beetles and mites breed alongside each other using the same carrion resource. We determined the fitness costs to beetles of associating with mites by focusing on fecundity costs, and assuming that the transport costs of carrying mites are similar for small and large beetles. We analyzed the effect of the mites on burying beetle reproductive success by giving beetles a mouse to breed upon, uncontested (see Methods section). We compared the number of larvae produced by small and large female beetles that carried either 30 mites or carried no mites at the onset of reproduction. The effect of mites differed with beetle size (GLMM, beetle size × mite treatment *χ*² = 4.27, df = 1, *P =* 0.039; Fig. 4B). Mites reduced the brood size of large beetles (GLMM, presence vs. absence of mites: *χ*² = 5.7, df = 1, *P =* 0.017; Fig. 4B) but they had no equivalent effect on small beetles (GLMM, presence vs. absence of mites: *χ*² = 0.09, df = 1, *P =* 0.763; Fig. 4B).

### ARE MITES MUTUALISTS OR PARASITES?

We used these experimental data to calculate the net effect of mites on the fitness of large and small beetles using the number of larvae produced as a measure of fitness. We multiplied the probability that the female would obtain a carcass in a contest (using data shown in Fig. 4A) by the mean number of larvae she would produce when breeding either with 30 mites or with no mites at all (using data in Fig. 4B). The calculations revealed that on average, 30 mites enhance the fitness of small loser female beetles, whereas they reduce the fitness of large winner females (Fig. 4C). Competitive interactions within burying beetles thus define a class of individuals for whom mites are mutualists, and a distinct class for whom the mites are parasites. Variation in competitive ability is, in turn, largely due to variation in adult body size which, we have previously shown, is strongly influenced by social and nutritional conditions experienced during development (Jarrett et al. 2017). Therefore, an individual's early life environment can predict whether its interactions with a partner species are mutualistic or parasitic.

### PARTNER CHOICE: WHO CHOOSES WHOM?

If beetles could choose how many mites to carry, then our results suggest that large beetles should prefer to carry none, whereas smaller beetles should prefer to carry 30 or more mites. Yet this is not the mite distribution we observe in natural populations (Fig. S3). Nor are we aware of any evidence that beetles can choose either to associate themselves with mites or to rid themselves of them. Indeed, other than the risk to larger beetles of reduced brood size when carrying mites, there appears to be no selection pressure to deter mites, particularly in the case of smaller beetles (Fig. 4C). However, previous work has shown that mites can choose their beetle partner (e.g., Grossman and Smith 2008). Therefore to understand the natural distribution of mite density per beetle, we considered the mites’ perspective in a final experiment.

We found that mite disembarkation onto the carrion takes place over a period of hours, and is thus completed long after the outcome of any contest over the carrion is decided (on average 29.19 ± 0.32 mites (97.29%) remained attached to the host beetle until a fight was won). This implies that mites are most likely to breed on carrion located by their beetle hosts, if their host has also secured ownership of the carrion (by winning a fight, for example). Even then, a further factor limiting individual mite reproductive success is the relative density of conspecifics. Previous work has shown that at high mite densities, the majority of deutonymphs do not molt into adults after acquiring a carcass, and therefore do not breed (Nehring and Müller 2009). We manipulated mite density, using the same approach as Nehring and Müller (2009) (see Methods section), and measured the probability that mites molted in each treatment. We found that mites were most likely to molt when carried in groups of 10, whereas groups of 30 were least likely to molt into adults (GLM, number of mites: *χ*² = 104.54, df = 2, *P* < 0.001; Fig. S7).

From the perspective of each individual mite carried on the beetle, the optimal mite density is therefore critically dependent on two factors that are unpredictable, and which have opposing effects on mite fitness. These are the likelihood that the host beetle can secure ownership of a carcass, which can be increased if mites travel at high densities, and the likelihood of molting to reproduce, once a carcass is secured, which is reduced if mites travel at high densities. Our data suggest that the distribution of mites that we observe in natural populations might be due to an adaptive balance between the costs and benefits of mites associating at high densities with beetles (see Methods section; Fig. S3). Or it may be that these costs and benefits are too unpredictable for mites to act strategically and that mite density is instead determined at random.

### CONCLUSION

Our experiments show that mites and beetles are sometimes in a shared‐benefit by‐product mutualism, in which they work together to secure a resource they both require for reproduction (Hoeksema and Bruna 2015). However, the extent of this interspecific mutualism is size dependent for beetles and density dependent for both species. In different ways, competitive interactions within burying beetles, and within mites, critically determine the fitness benefits that can be gained from interacting with the other species. Conspecifics thus play a key role in determining when mutualisms between species will persist and when they are likely to degrade into more antagonistic interactions. For small beetles, a competitive disadvantage against conspecific rivals turns mites into mutualists, though they are parasites for larger beetles. Furthermore, competition among mites for the opportunity to breed means that although small beetles potentially benefit from transporting mites at high densities, on average the mites themselves have greater fitness when traveling at lower densities. As mites can choose their beetle host, and beetles apparently do not choose their mites, in natural populations relatively few small beetles carry a sufficiently high density of mites for them to be mutualists.

Associate Editor: A. Gardner

## Supporting information


**Figure S1**. A burying beetle *N. vespilloides* bearing mites from the *P. carabi* species complex.

**Figure S2**. Frequency distribution of burying beetle body size, given by pronotum width, of wild‐caught *N. vespilloides* across three woodlands in Cambridgeshire.

**Figure S3**. (a) Frequency distribution of the number of mites carried, for all wild‐caught *N. vespilloides*, and (b) the number of mites carried by small and large wild‐caught *N. vespilloides*.

**Figure S4**. The relationship between mite load and beetle body temperature, relative to soil temperature.

**Figure S5**. The percentage increase in body temperature as a consequence of carrying mites in relation to body size, given by pronotum width.

**Figure S6**. (a) Effect of mite density on the proportion of beetle surface area covered, relative to its size and (b) the size of the uninsulated area that remains, relative to the beetle's size.

**Figure S7**. Effect of mite density on mite reproductive success.

**Table S1**. Results from the models analyzing changes in body temperature as a function of the load carried by beetles during the treadmill experiments for small and large beetles.

**Supplementary Video 1**. A beetle with mites attacking a beetle without mites. Inset is the corresponding thermal imaging video.

**Supplementary Video 2**. A beetle walking on a motorized treadmill.
