# Supplementary figures and images for: Conflict within species determines the value of a mutualism between species
Source: Evol Lett. 2019 Mar 6;3(2):185–97. doi: 10.1002/evl3.109 (PMC6457395; doi:10.1002/evl3.109)

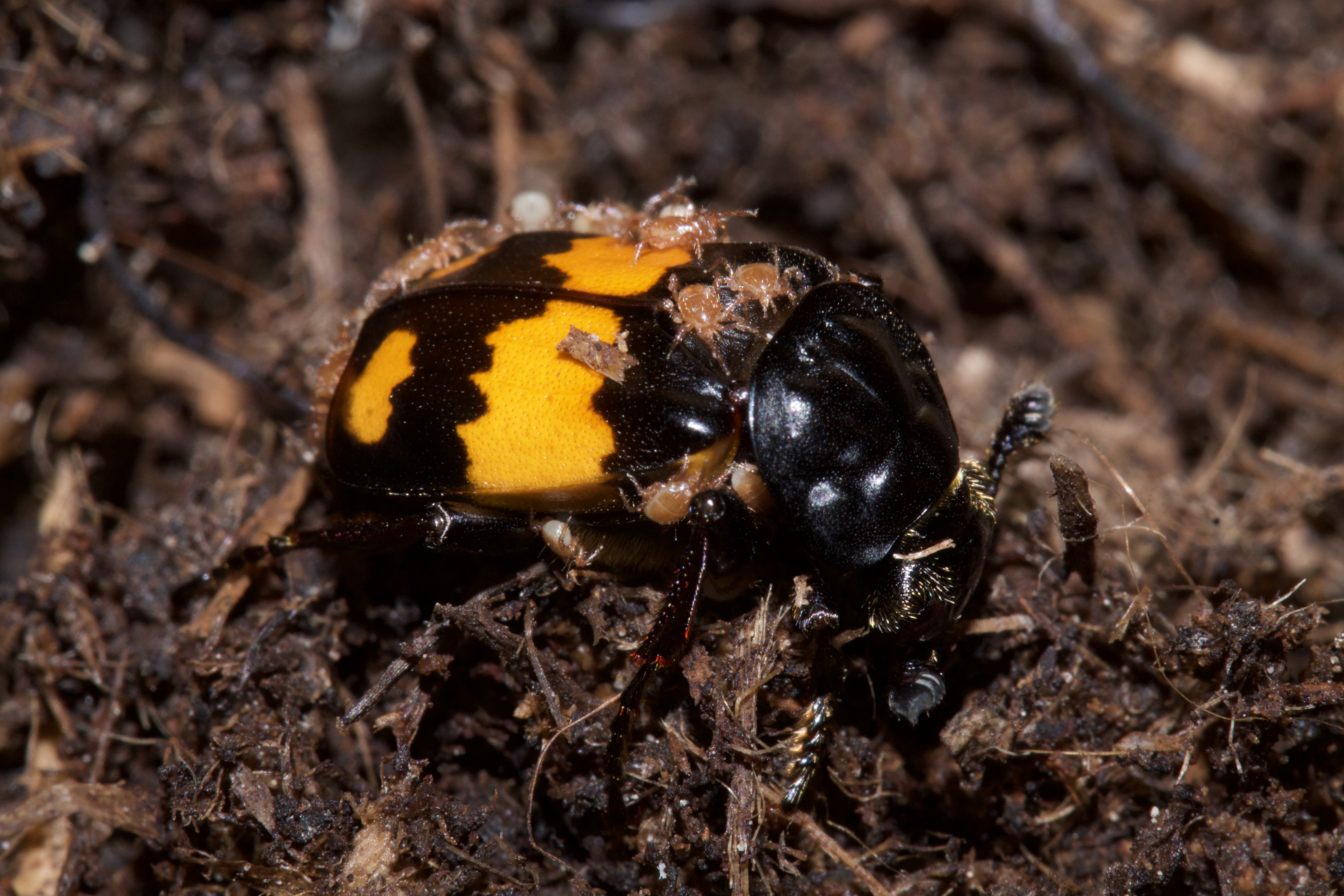

Supplement: Supplementary file 1 — Figure S1. A burying beetle N. vespilloides bearing mites from the P. carabi species complex. [file EVL3-3-185-s001.jpg]

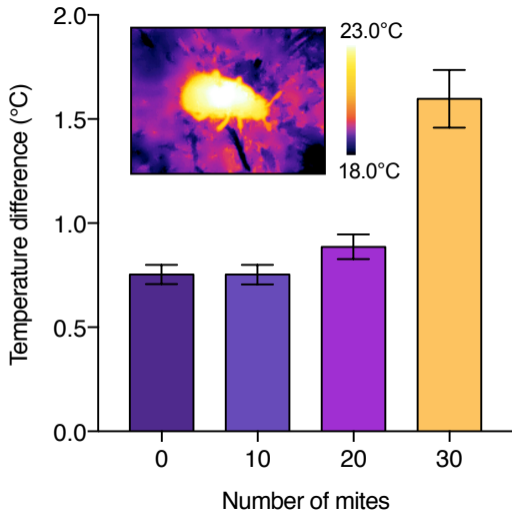

Supplement: Supplementary file 4 — Figure S4. The relationship between mite load and beetle body temperature, relative to soil temperature. [file EVL3-3-185-s004.pdf]

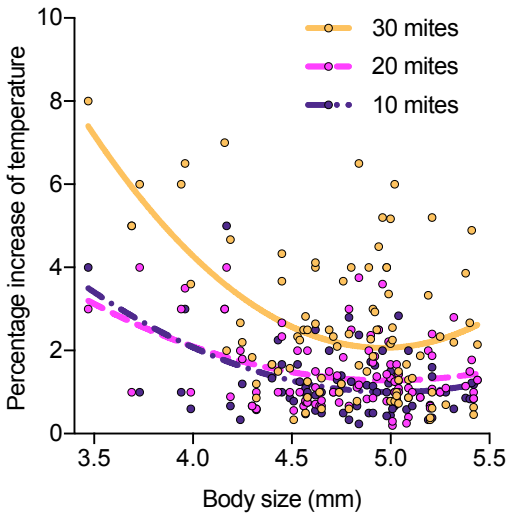

Supplement: Supplementary file 5 — Figure S5. The percentage increase in body temperature as a consequence of carrying mites in relation to body size, given by pronotum width. [file EVL3-3-185-s005.pdf]

Proportion of mites moulted

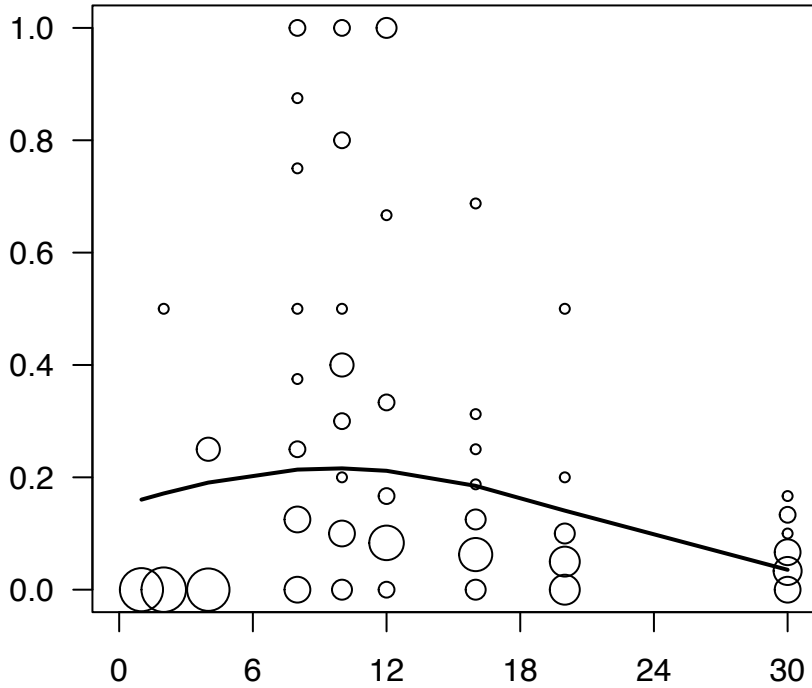

Number of mites

Supplement: Supplementary file 7 — Figure S7. Effect of mite density on mite reproductive success. [file EVL3-3-185-s007.pdf]
