# Supplementary material for: Conflict within species determines the value of a mutualism between species
Source: Evol Lett. 2019 Mar 6;3(2):185–97. doi: 10.1002/evl3.109 (PMC6457395; doi:10.1002/evl3.109)
Supplement: Supplementary file 8 — Table S1. Results from the models analyzing changes in body temperature as a function of the load carried by beetles during the treadmill experiments for small and large beetles. [file EVL3-3-185-s008.docx]

**Table S1 | Results from the models analysing changes in body temperature as a function of the load carried by beetles during the treadmill experiments, for small and large beetles.**

| ***Small beetles*** |  |  |  |  |  |  |
| --- | --- | --- | --- | --- | --- | --- |
| Response variables | Temperature difference (walking) | | | Temperature difference (resting) | | |
| Independent variables | 𝑥^2^ | d.f. | *p*-value | 𝑥^2^ | d.f. | *p*-value |
| Intercept | 0.00 | 1 | 1.000 | 0.00 | 1 | 1.000 |
| Loading treatments | 0.00 | 2 | 1.000 | 0.00 | 2 | 1.000 |
| **Time** | **65.58** | **6** | **<0.001** | **68.62** | **9** | **<0.001** |
| **Loading treatments*Time** | **23.35** | **12** | **0.025** | **61.34** | **18** | **<0.001** |
|  |  |  |  |  |  |  |
| ***Large beetles*** |  |  |  |  |  |  |
| Response variables | Temperature difference (walking) | | | Temperature difference (resting) | | |
| Independent variables | 𝑥^2^ | d.f. | *p*-value | 𝑥^2^ | d.f. | *p*-value |
| Intercept | 0.00 | 1 | 0.963 | 0.086 | 1 | 0.770 |
| Loading treatments | 4.53 | 2 | 0.104 | 2.66 | 2 | 0.265 |
| **Time** | **462.70** | **6** | **<0.001** | 11.51 | 9 | 0.242 |

*After detecting a significant three-way beetle size x treatment x time interaction, the dataset was split by beetle body size. The dependent variables were the temperature difference (walking: focal body temperature – body temperature at time 0 s; resting: focal body temperature – body temperature at time 60 s). Loading treatments (control, mite, and weight) were included as independent variables and beetle identity was included as a random factor. Significant factors are shown in bold.
